# Reimagining Data for Equity: Reaching the Unreached Map Explorer for Localized Public Health Intelligence in the Western Pacific Region

**DOI:** 10.2196/93324

**Published:** 2026-08-26

**Authors:** Sumbul Hashmi, Anne-Laure Budts, Vincent Meurrens, Jessica Sarah Stow, Fukushi Morishita, Rajendra-Prasad Yadav

**Affiliations:** 1 EPCON, Antwerp, Belgium; 2 EPCON, Workshop 17 146 Campground Rd, 3rd floor, Snakepit Building, Newlands, Cape Town, Western Cape, 7780, South Africa, +63 2 8528 8001; 3 World Health Organization Regional Office for the Western Pacific, Manila, Philippines

**Keywords:** geospatial intelligence, digital health, vulnerability mapping, public health surveillance, health equity

## Abstract

Geographic information systems (GISs) and digital dashboards have become central to epidemic monitoring and crisis response, yet their broader potential for routine, equity-focused public health decision-making remains underused. As health systems increasingly prioritize reaching underserved and vulnerable populations, mapping platforms must evolve from static visualization tools to dynamic decision-support systems. This paper reflects on the development and strategic role of the Reaching the Unreached (RTU) map explorer, a regional geospatial decision-intelligence platform developed in collaboration with the World Health Organization Regional Office for the Western Pacific. The RTU map explorer integrates high-resolution subnational data across 36 countries and areas in the Western Pacific Region. It consolidates demographic, socioeconomic, health access, and vulnerability indicators into a unified geoportal that enables visualization at resolutions ranging from the national level to the village level. Drawing on open-access spatial repositories and modeled datasets, the platform applies population-based spatial clustering methods to settings where administrative units are too large to capture meaningful local variation. Built using an open-source architecture, the platform offers interactive functionalities, including layer activation, filtering, prioritization tools, and location-specific mapping, to support localized planning and resource allocation. By combining contextual and health-related data within a single interface, the RTU map explorer facilitates a shift from reactive surveillance to proactive, equity-oriented decision-making. Policymakers can compare vulnerabilities across regions, program managers can prioritize high-need districts or communities, and local implementers can identify specific locations for targeted interventions. Although challenges remain, including data recency, modeled data uncertainty, and the need for field validation, the platform illustrates how integrated geospatial intelligence can move public health beyond documenting disparities to actively addressing them through localized, data-driven action.

## Introduction

Geographic information systems (GISs) and mapping platforms have long been used in public health, with the most frequent application being epidemic monitoring and disease surveillance [1,2]. A near-global use of such tools was observed during the COVID-19 pandemic, during which mapping platforms and live dashboards provided intuitive outputs and were extensively leveraged [3]. However, as the epidemic curve plateaus, the use of these highly valuable applications also tends to diminish [4]. Such solutions possess considerable potential to address numerous public health questions and support routine decision-making when properly implemented [3]. These tools are beneficial for a range of stakeholders, including policymakers, program managers, and local implementers such as community-based organizations.

To ensure a map’s utility for all stakeholders and its relevance to routine decision-making, the required spatial resolution must transcend the national or district level [5]. A well-designed visualization can address critical questions such as the following: “Which villages lack basic Water, Sanitation, and Hygiene (WASH) services?” “Where are pregnant women least likely to deliver in a maternity facility?” “Where are children dropping out of school?” “Where do children require additional nutritional assistance?” “Which areas have poor connectivity to primary healthcare?” or “Which communities are lagging in childhood immunization coverage?” [6]. Answering these questions requires a comprehensive understanding of the local context; therefore, integrating this information into a single platform is a logical approach [7].

## The Map Explorer for Reaching the Unreached: A Regional Decision-Intelligence Platform

One such platform, the Reaching the Unreached (RTU) map explorer for the Western Pacific Region, encompasses 36 countries and areas within the World Health Organization (WHO) Western Pacific Region. Indonesia, which transitioned from the WHO South-East Asia Region to the Western Pacific Region in May 2025, was not included because all analyses were completed prior to this reassignment. The Pitcairn Islands were also excluded due to their small population size. This paper reflects on our experience developing the platform, which is a regional initiative by the WHO to strengthen data-driven decision-making. We discuss its evolution as a regional intelligence tool and how it can support the transition toward localized, equitable, and continuous decision-support systems in public health. The platform was designed for use by regional, national, and subnational public health decision-makers, including WHO teams, ministries of health, program managers, funding partners, and nongovernmental or community-based implementers seeking to identify underserved populations and guide more localized, equitable action.

The platform was developed by EPCON in September 2024 in collaboration with the WHO Regional Office for the Western Pacific to support the implementation of the WHO Regional Framework for RTU [8]. The overarching goal of this project was to develop a platform capable of supporting data-driven decision-making for common public health concerns and of assisting in the identification of vulnerable populations at scales extending down to the village or community level.

The RTU map explorer hosts high-resolution, subnational data spanning a range of indicators, including demographic characteristics, socioeconomic development, overall human development, health care access, and population vulnerability. The data are available across all administrative levels, from the national level down to a resolution of a few kilometers (village or neighborhood level). This granular data make the platform suitable for diverse public health programs, such as those focused on disease control (eg, tuberculosis [TB], HIV, malaria, and other epidemics) as well as programs related to population welfare, education, immunization, WASH, and health promotion, with a focus on underserved or unreached populations [9,10]. Given the constraints of limited resources and a high vulnerability burden, the efficient use of resources is paramount [11]. Investing in advanced tools that facilitate context-driven decision-making can ensure that interventions reach the populations most in need and optimize intervention implementation [12].

The RTU map explorer is built on a geoportal, the web-based mapping component of the Epi-control platform (EPCON). The Epi-control platform is an AI-driven “digital twin” that is deployed in several countries to support specific disease control programs, primarily TB and HIV [13,14]. Within the Epi-control platform, data from health programs can be augmented with high-resolution subnational contextual data to train predictive models that identify high-transmission areas or disease “hotspots.” The “digital twin” is a dynamic model that updates in near real-time, or as required, to forecast new hot spots based on programmatic progress and planning. Community-based interventions are guided by a comprehensive, data-driven process, moving beyond sole reliance on case notification rates. This approach has yielded improved intervention outcomes and helped direct resources to priority areas, thereby maximizing program impact [14,15]. However, the implementation of this approach and its outcome evaluation in the Western Pacific Region have yet to be evaluated. The RTU map explorer functions as a data integration and visualization platform, providing the static contextual intelligence layer upon which, when combined with programmatic or case notification data, programmatic decision-making can be grounded.

## Transforming the Role of Maps

The first step was a thorough search for subnational high-resolution data. Relevant data sources were identified from open online repositories that host survey or modeled indicators, including the Demographic and Health Surveys (DHS) spatial repository, the Institute for Health Metrics and Evaluation (IHME), WorldPop, the Humanitarian Data Exchange (HDX), and country government websites. The data sources were selected based on the criteria of resolution, recency, and extent; a complete inventory is provided in Multimedia Appendix 1. Such high-resolution raster data were extracted and aggregated within each administrative boundary using zonal statistics. Pixel values were either summed (for count-based data, eg, population counts) or averaged (for continuous or rate-based data, eg, prevalence data). To aggregate values up the administrative hierarchy, population-weighted averages were used for indicators that vary with population size (eg, vaccination coverage), and mean values were used for those that do not vary with population size (eg, distance to the nearest health care facility).

On the basis of the availability of high-resolution data and high population density, the smallest administrative units in some countries were further subdivided into smaller units. This was achieved using a recursive weighted k-means clustering algorithm [16]. For each administrative unit, the algorithm iteratively assigned population-weighted centroids using k-means clustering, from which “Thiessen” (Voronoi) polygons were constructed and their populations computed [17]. If the population of a generated polygon exceeded a target threshold of approximately 10,000 individuals, the unit was iteratively subdivided. The 10,000-person population threshold was determined in collaboration with project stakeholders to make administrative units comparable across the 36 countries and areas of the Western Pacific Region.

The weighted k-means algorithm partitioned the population into clusters, after which new Voronoi polygons were generated around the resulting cluster centroids, and oversized polygons were replaced with these smaller units. This process was repeated recursively until all polygons fell within the target population range. Each polygon was then spatially clipped to fit within its original administrative boundary. The final output comprised a table of Thiessen polygons, each associated with a population count and clipped geometry. The map explorer is implemented using proprietary Python code (version 3.12.3; Python Software Foundation) and leverages open-source software components.

**Figure 1.**
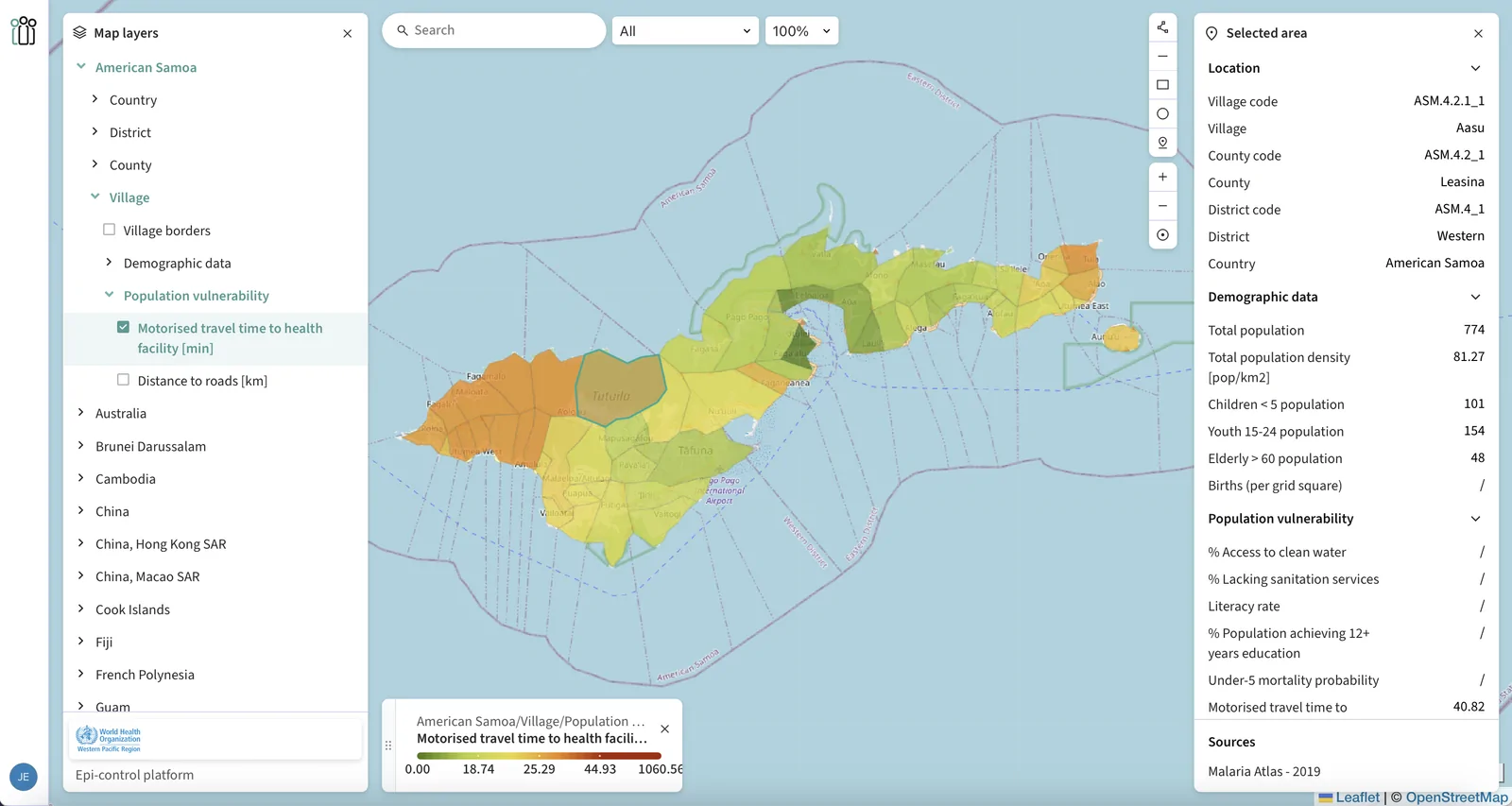
Arrangement of data layers for American Samoa, showing motorized travel time to health facilities at the village level. The color scale ranges from green (low travel time to health facilities) to orange red (high travel time to health facilities).

The data layers are organized separately for each of the 36 countries and areas, followed by a comprehensive “Western Pacific Region” tab that visualizes all countries simultaneously for intercountry comparisons. For each country, the data layers are subdivided based on the respective administrative levels, commencing at the national level, followed by district, county, and village levels. Figure 1 demonstrates the selection of a layer to visualize data at the highest available resolution for American Samoa (village).

**Figure 2.**
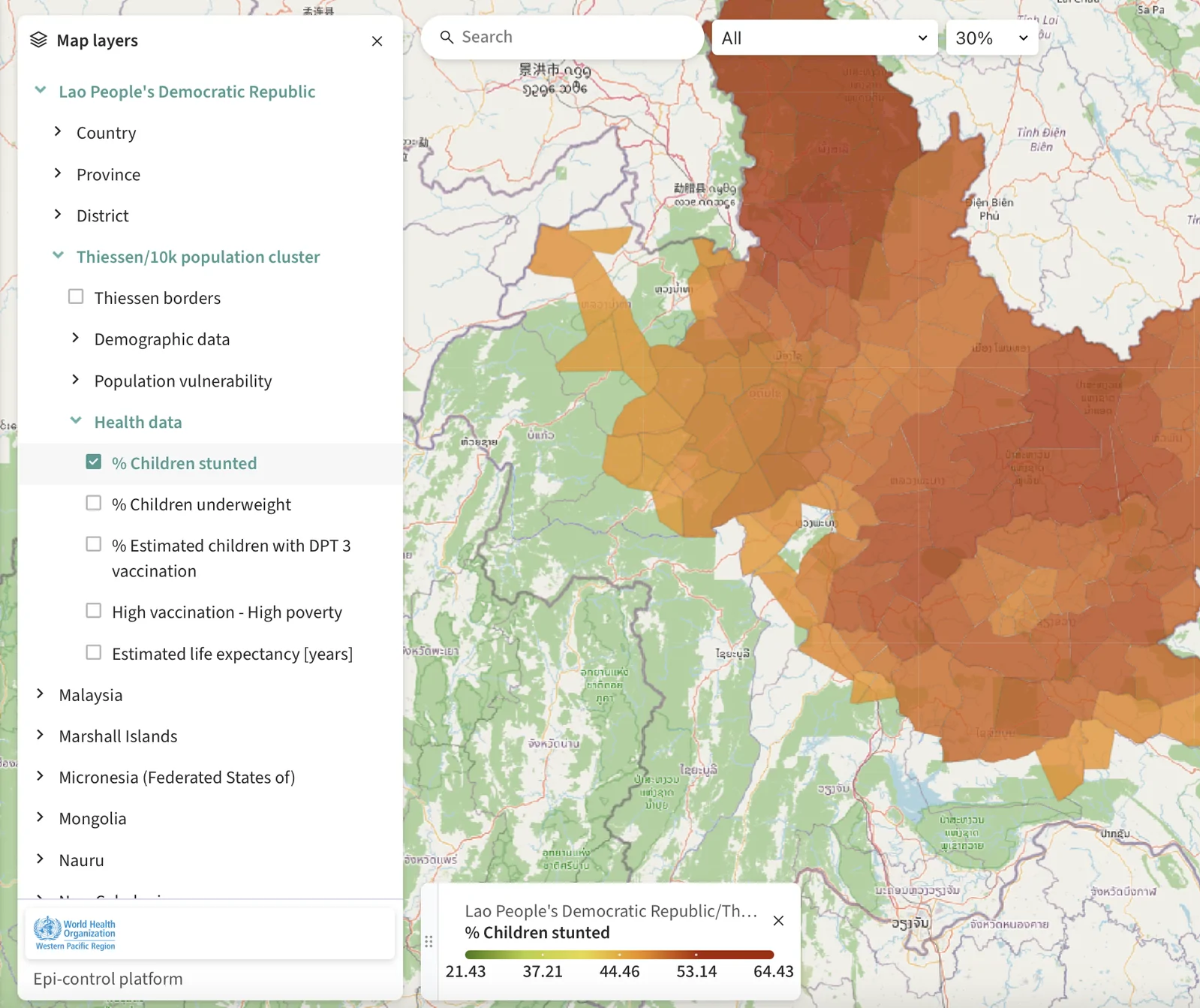
Enlarged view of the data layers available under different categories at the Thiessen-level (subdistrict-level) visualization of children stunting prevalence in the Lao People’s Democratic Republic. The values are filtered to display the top 30% Thiessens with the highest prevalence of child stunting. The color scale ranges from green (low percentage of children stunted) to orange red (high percentage of children stunted). Thiessen borders are shown in lighter lines, and active layers are displayed in green on the left-side layer panel.

The map explorer provides several functionalities for seamless exploration of data layers. To commence exploration of a particular country, a data layer must be selected to activate the map. The “focus” tool facilitates direct zooming onto the country whose layer has been activated. The “location pointer” tool allows users to place a “pin” directly on the map and provides a Google Maps link to that specific location. This link can be shared or used to explore the neighborhood within the village or community, proving particularly useful for local implementers or nongovernmental organizations (NGOs) working closely with communities. It aids in identifying a specific landmark or village community for outreach purposes. The “filter” option enables the shortlisting of priority locations for targeted interventions. Figure 2 demonstrates the filtering capability of the platform to identify at-risk communities with high prevalence of child stunting. This is valuable for both program management and community-based organizations, allowing users to filter priority districts, wards, villages, or communes for specific resource allocation or intervention planning.

## Bridging the Gap Between Data and Decision

The platform is designed to support public health decision-making, the planning of local programs, and disease surveillance. The extensive data library can facilitate horizontal program integration. Sociodemographic conditions and local context are determinants not only of health and disease conditions but also of human behavior, local practices, and the design of interventions [18]. The platform offers an opportunity to gain a comprehensive understanding of the local context from the national to the village level, making it suitable for designing integrated programs such as TB and nutritional support interventions, maternal and child health (MCH) and immunization programs, and education and financial support initiatives.

**Figure 3.**
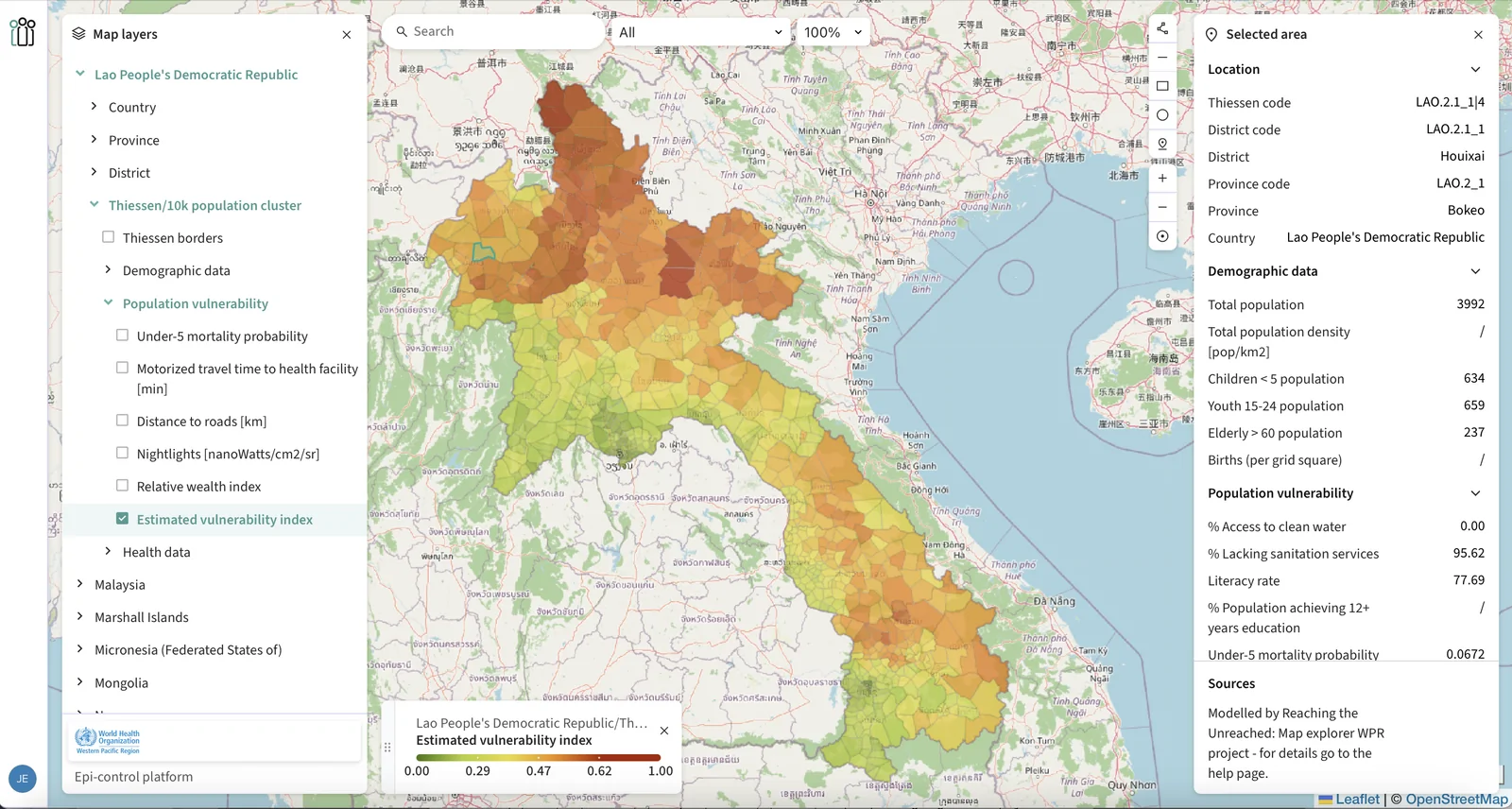
Thiessen-level (subdistrict-level) visualization of estimated vulnerability in the Lao People’s Democratic Republic. The color scale ranges from green (low vulnerability) to orange red (high vulnerability).

**Figure 4.**
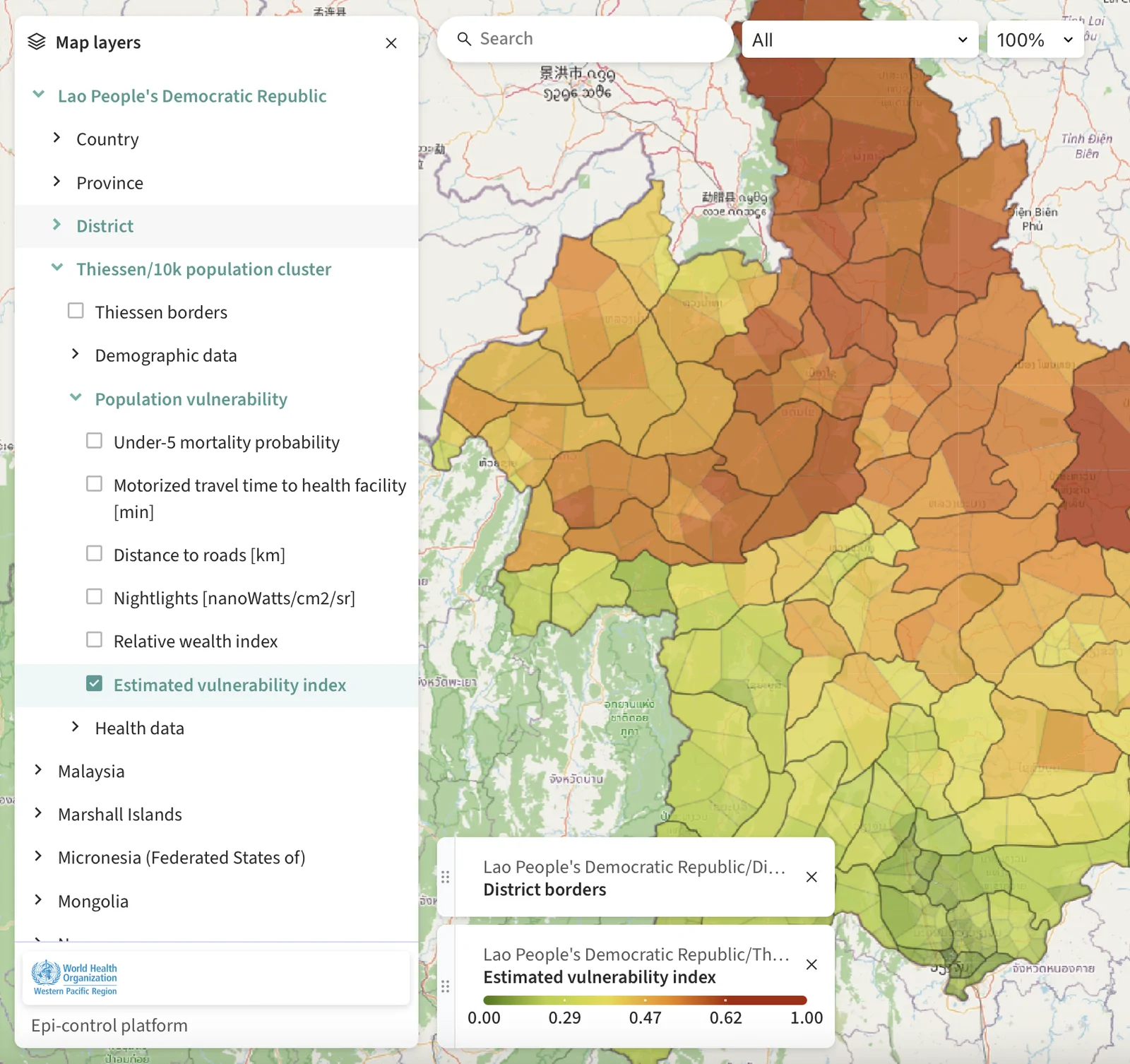
Enlarged view of the Thiessen-level (subdistrict-level) visualization of estimated vulnerability in the Lao People’s Democratic Republic. The color scale ranges from green (low vulnerability) to orange red (high vulnerability). District borders are shown in dark lines, and Thiessen borders are shown in lighter lines.

To further support decision-making across multiple administrative units, an estimated vulnerability index was constructed using 11 indicators spanning 3 domains: health access (travel time to the nearest health center, distance to roads, and vaccination coverage), demographic and health outcomes (population density, elderly population aged ≥60 years, child mortality, and the prevalence of underweight children), and socioeconomic conditions (male and female literacy, nightlight intensity, access to water, and the prevalence of open defecation). For each indicator, spatial units were ranked relative to one another, with the direction of ranking assigned so that a higher rank consistently represented greater vulnerability. Each unit’s rank was then normalized by dividing it by the maximum rank observed for that indicator, producing a score between 0 and 1 for every indicator across all spatial units. The vulnerability index for each spatial unit was computed as the mean of its normalized ranks across all available indicators. This formulation treats each indicator with equal weight and accommodates spatial units with missing data by computing the mean over observed indicators only, ensuring that partial data does not systematically bias the index. Figures 3 and 4 demonstrate the visualization of the vulnerability index at high resolution (Thiessen). For visualization at less granular administrative levels, unit-level index scores were spatially aggregated using the median to ensure robustness to outlying values within administrative units. The resulting index is a relative measure of estimated vulnerability, situating each administrative unit within the national distribution of vulnerability across all 3 domains simultaneously. The index is intended to support exploratory prioritization and dialogue with countries, not as a resource-allocation tool.

The mapping of data across all administrative levels ensures the platform’s suitability for stakeholders across multiple professional cadres. Policy-level stakeholders may require a high-level overview, program implementers may need more granular information at the community level, and the scientific community may require both. Access levels and the scope of data can be customized based on local requirements, balancing comprehensiveness with simplicity.

## Toward a More Equitable Data Future

The RTU map explorer is designed to support near real-time data updates when linked to a country’s data platform or disease notification system, allowing new cases, interventions, or planned activities to be dynamically mapped on the platform. This integration is facilitated on a case-by-case basis according to country needs and data infrastructure. This also supports monitoring and evaluation efforts and contributes to pandemic preparedness strategies. As new survey data or modeled estimates become available, the data sources can be refreshed. For instance, the platform is currently being used in other countries, such as Nigeria [15], Pakistan [14], Uganda [19], and the Philippines, for planning community-based TB active case-finding interventions. Program stakeholders are able to track progress and plan interventions based on their access level.

Although the RTU map explorer platform has yet to achieve full uptake in the region, it has been integrated in other countries with mobile apps, diagnostic tools [20], X-ray equipment, and District Health Information Software 2 (DHIS2) platforms to access and map health-related data in real-time or to transmit notifications and data to other dashboards or devices.

Although various open-source platforms make subnational high-resolution data publicly available, this map explorer is unique in integrating data from different domains into a single platform, thereby maximizing its potential for scientific, policy-level, and programmatic decision-making. Mapping and granular visualizations illuminate details that would remain obscured within aggregated numbers in tables and graphs, which is particularly crucial when attempting to reach underserved, remote, and vulnerable population groups [21]. The mapping of the local context enhances data intuitiveness and fosters opportunities for new ideas, approaches, and discussions [22].

In its current form, the platform is beneficial for government agencies, funding organizations, and local implementers. Funding agencies can use it to compare vulnerable regions both across and within countries to identify resource needs. Program planners and ministries can use it to identify high-priority regions within their respective countries. Local organizations can pinpoint communities or neighborhoods for targeted interventions, informed by the backdrop of local contextual data.

However, maintaining contextual and demographic data sources on such a platform comes with certain challenges. Reliable estimates and granular data may not be available for all countries and regions. These estimates are not updated on fixed timelines; therefore, the most recent granular estimates can be ≥5 years old for some countries. Second, most granular estimates are modeled datasets, which can inherently have large uncertainties. The platform in its current state is unable to visualize the associated CIs or uncertainties for these modeled estimates. The platform is not currently open source and comes with a licensing fee. The governance framework for platform adoption, including country-specific data-sharing, access, and stewardship arrangements, will be an important consideration when nonpublicly available data are incorporated into the platform. For a more tailored experience and to facilitate joint efforts in programmatic decision-making, the subsequent phase would involve the creation of individual mapping portals for specific countries. There is also a need for field validation and evaluation of the feasibility and uptake of the platform in the Western Pacific Region.

Data can generate greater impact when they empower those closest to the problem. Public health systems need to look beyond documenting inequality; there is a need for active tools that help make decisions. The progress of public health intelligence will depend not only on accumulating more data but also on transforming existing data into meaningful, local, relevant, and equitable insights. The RTU map explorer exemplifies technology that supports decision-making that is not just more intelligent, but also fairer.

## Supplementary material

10.2196/93324 Multimedia Appendix 1 List of data sources used to set up the platform.

## Abbreviations

DHIS2: District Health Information Software 2
DHS: Demographic and Health Surveys
GIS: geographic information system
HDX: Humanitarian Data Exchange
IHME: Institute for Health Metrics and Evaluation
MCH: maternal and child health
NGO: nongovernmental organization
RTU: Reaching the Unreached
TB: tuberculosis
WASH: water, sanitation, and hygiene
WHO: World Health Organization
