# Supplementary material for: Reimagining Data for Equity: Reaching the Unreached Map Explorer for Localized Public Health Intelligence in the Western Pacific Region
Source: JMIR Public Health Surveill. 2026 Aug 26;12:e93324. doi: 10.2196/93324 (PMC13513218; doi:10.2196/93324)
Supplement: Supplementary file 1 [file publichealth-v12-e93324-s001.pdf]

Country name	Data source type	Indicator
American Samoa	Indicators of human development	Gridded Relative Deprivation Index
American Samoa	Administrative boundaries	Distance to road in kilometers
American Samoa	Demographic data	Total population aged greater than 60 years
American Samoa	Environmental covariates	Nightlights in nanoWatts per centimeter squared per steradian
American Samoa	Demographic data	Total population
American Samoa	Demographic data	Population density of the total population per kilometers squared
American Samoa	Health related data	Motorized travel time to a healthcare facility in minutes
American Samoa	Demographic data	Total population aged less than 5 years
American Samoa	Demographic data	Total population aged in the range of 15 to 24 years
Australia	Indicators of human development	Gridded Relative Deprivation Index
Australia	Administrative boundaries	Distance to road in kilometers
Australia	Demographic data	Total population aged greater than 60 years
Australia	Indicators of human development	Mortality rate of children under 5 years
Australia	Environmental covariates	Nightlights in nanoWatts per centimeter squared per steradian
Australia	Indicators of human development	Percentage of children experiencing underweight/malnutrition
Australia	Demographic data	Total population
Australia	Demographic data	Population density of the total population per kilometers squared
Australia	Health related data	Motorized travel time to a healthcare facility in minutes
Australia	Demographic data	Total population aged less than 5 years
Brunei Darussalam	Indicators of human development	Gridded Relative Deprivation Index
Brunei Darussalam	Demographic data	Births per grid square
Brunei Darussalam	Demographic data	Total population aged in the range of 15 to 24 years
Brunei Darussalam	Administrative boundaries	Distance to road in kilometers
Brunei Darussalam	Demographic data	Total population aged greater than 60 years
Brunei Darussalam	Indicators of human development	Literacy proportion

Country name	Data source type	Indicator
Brunei Darussalam	Environmental covariates	Nightlights in nanoWatts per centimeter squared per steradian
Brunei Darussalam	Demographic data	Total population
Brunei Darussalam	Demographic data	Population density of the total population per kilometers squared
Brunei Darussalam	Health related data	Motorized travel time to a healthcare facility in minutes
Brunei Darussalam	Demographic data	Total population aged less than 5 years
Australia	Demographic data	Total population aged in the range of 15 to 24 years
Cambodia	Indicators of human development	Distance to road in kilometers
Cambodia	Indicators of human development	Nightlights in nanoWatts per centimeter squared per steradian
Cambodia	Indicators of human development	Percentage of children who have received a measles vaccination
China	Indicators of human development	Gridded Relative Deprivation Index
China	Demographic data	Births per grid square
China	Administrative boundaries	Distance to road in kilometers
China	Indicators of human development	International wealth index
China	Demographic data	Life expectancy at birth
China	Indicators of human development	Literacy rate
China	Indicators of human development	Literacy proportion
China	Indicators of human development	Literacy rate of women
China	Environmental covariates	Nightlights in nanoWatts per centimeter squared per steradian
China	Indicators of human development	Percentage of children experiencing underweight/malnutrition
China	Indicators of human development	Percentage of children experiencing stunted development
China	Demographic data	Total population
China	Demographic data	Population density of the total population per kilometers squared
China	Health related data	Motorized travel time to a healthcare facility in minutes
Cook Islands	Indicators of human development	Gridded Relative Deprivation Index
Cook Islands	Administrative boundaries	Distance to road in kilometers

Country name	Data source type	Indicator
Cook Islands	Environmental covariates	Nightlights in nanoWatts per centimeter squared per steradian
Cook Islands	Health related data	Motorized travel time to a healthcare facility in minutes
Fiji	Indicators of human development	Gridded Relative Deprivation Index
Fiji	Administrative boundaries	Distance to road in kilometers
Fiji	Demographic data	Total population aged greater than 60 years
Fiji	Indicators of human development	International wealth index
Fiji	Indicators of human development	Mortality rate of children under 5 years
Fiji	Environmental covariates	Nightlights in nanoWatts per centimeter squared per steradian
Fiji	Demographic data	Total population
Fiji	Demographic data	Population density of the total population per kilometers squared
Fiji	Health related data	Motorized travel time to a healthcare facility in minutes
Fiji	Demographic data	Total population aged less than 5 years
Fiji	Demographic data	Total population aged in the range of 15 to 24 years
French Polynesia	Indicators of human development	Gridded Relative Deprivation Index
French Polynesia	Administrative boundaries	Distance to road in kilometers
French Polynesia	Environmental covariates	Nightlights in nanoWatts per centimeter squared per steradian
French Polynesia	Health related data	Motorized travel time to a healthcare facility in minutes
Guam	Indicators of human development	Gridded Relative Deprivation Index
Guam	Demographic data	Total population aged greater than 60 years
Guam	Environmental covariates	Nightlights in nanoWatts per centimeter squared per steradian
Guam	Demographic data	Total population
Guam	Demographic data	Population density of the total population per kilometers squared
Guam	Health related data	Motorized travel time to a healthcare facility in minutes
Guam	Demographic data	Total population aged less than 5 years
Guam	Administrative boundaries	Distance to road in kilometers

Country name	Data source type	Indicator
Guam	Demographic data	Total population aged in the range of 15 to 24 years
China, Hong Kong SAR	Indicators of human development	Gridded Relative Deprivation Index
China, Hong Kong SAR	Administrative boundaries	Distance to road in kilometers
China, Hong Kong SAR	Demographic data	Life expectancy at birth
China, Macao SAR	Demographic data	Life expectancy at birth
China, Hong Kong SAR	Environmental covariates	Nightlights in nanoWatts per centimeter squared per steradian
China, Hong Kong SAR	Health related data	Motorized travel time to a healthcare facility in minutes
Japan	Indicators of human development	Gridded Relative Deprivation Index
Japan	Administrative boundaries	Distance to road in kilometers
Japan	Demographic data	Total population aged greater than 60 years
Japan	Environmental covariates	Nightlights in nanoWatts per centimeter squared per steradian
Japan	Demographic data	Total population
Japan	Demographic data	Population density of the total population per kilometers squared
Japan	Health related data	Motorized travel time to a healthcare facility in minutes
Japan	Demographic data	Total population aged less than 5 years
Japan	Demographic data	Births per grid square
Japan	Demographic data	Total population aged in the range of 15 to 24 years
Kiribati	Indicators of human development	Gridded Relative Deprivation Index
Kiribati	Demographic data	Total population aged greater than 60 years
Kiribati	Environmental covariates	Nightlights in nanoWatts per centimeter squared per steradian
Kiribati	Demographic data	Total population
Kiribati	Demographic data	Population density of the total population per kilometers squared
Kiribati	Health related data	Motorized travel time to a healthcare facility in minutes
Kiribati	Demographic data	Total population aged less than 5 years
Kiribati	Administrative boundaries	Distance to road in kilometers
Kiribati	Demographic data	Total population aged in the range of 15 to 24 years
Lao People's Democratic Republic (the)	Indicators of human development	Distance to road in kilometers

Country name	Data source type	Indicator
Lao People's Democratic Republic (the)	Indicators of human development	Nightlights in nanoWatts per centimeter squared per steradian
Lao People's Democratic Republic (the)	Indicators of human development	Percentage of children who have received a polio vaccination
China, Macao SAR	Indicators of human development	Gridded Relative Deprivation Index
China, Macao SAR	Administrative boundaries	Distance to road in kilometers
China, Macao SAR	Environmental covariates	Nightlights in nanoWatts per centimeter squared per steradian
China, Macao SAR	Demographic data	Total population
China, Macao SAR	Demographic data	Population density of the total population per kilometers squared
China, Macao SAR	Health related data	Motorized travel time to a healthcare facility in minutes
Malaysia	Indicators of human development	Distance to road in kilometers
Malaysia	Indicators of human development	Nightlights in nanoWatts per centimeter squared per steradian
China, Hong Kong SAR	Indicators of human development	DPT 3 coverage level
Marshall Islands	Indicators of human development	Gridded Relative Deprivation Index
Marshall Islands	Administrative boundaries	Distance to road in kilometers
Marshall Islands	Environmental covariates	Nightlights in nanoWatts per centimeter squared per steradian
Marshall Islands	Health related data	Motorized travel time to a healthcare facility in minutes
Micronesia (Federated States of)	Indicators of human development	Gridded Relative Deprivation Index
Micronesia (Federated States of)	Administrative boundaries	Distance to road in kilometers
Micronesia (Federated States of)	Demographic data	Total population aged greater than 60 years
Micronesia (Federated States of)	Environmental covariates	Nightlights in nanoWatts per centimeter squared per steradian
Micronesia (Federated States of)	Demographic data	Total population
Micronesia (Federated States of)	Demographic data	Population density of the total population per kilometers squared
Micronesia (Federated States of)	Health related data	Motorized travel time to a healthcare facility in minutes

Country name	Data source type	Indicator
Micronesia (Federated States of)	Demographic data	Total population aged less than 5 years
Micronesia (Federated States of)	Demographic data	Total population aged in the range of 15 to 24 years
Mongolia	Indicators of human development	Distance to road in kilometers
Mongolia	Indicators of human development	Nightlights in nanoWatts per centimeter squared per steradian
Mongolia	Indicators of human development	Percentage of children who have received a polio vaccination
Nauru	Indicators of human development	Gridded Relative Deprivation Index
Nauru	Administrative boundaries	Distance to road in kilometers
Nauru	Demographic data	Total population aged greater than 60 years
Nauru	Environmental covariates	Nightlights in nanoWatts per centimeter squared per steradian
Nauru	Demographic data	Total population
Nauru	Demographic data	Population density of the total population per kilometers squared
Nauru	Health related data	Motorized travel time to a healthcare facility in minutes
Nauru	Demographic data	Total population aged less than 5 years
Nauru	Demographic data	Total population aged in the range of 15 to 24 years
New Caledonia	Indicators of human development	Gridded Relative Deprivation Index
New Caledonia	Administrative boundaries	Distance to road in kilometers
New Caledonia	Demographic data	Total population aged greater than 60 years
New Caledonia	Environmental covariates	Nightlights in nanoWatts per centimeter squared per steradian
New Caledonia	Demographic data	Total population
New Caledonia	Demographic data	Population density of the total population per kilometers squared
New Caledonia	Health related data	Motorized travel time to a healthcare facility in minutes
New Caledonia	Demographic data	Total population aged less than 5 years
New Caledonia	Demographic data	Total population aged in the range of 15 to 24 years
New Zealand	Indicators of human development	Gridded Relative Deprivation Index
New Zealand	Administrative boundaries	Distance to road in kilometers

Country name	Data source type	Indicator
New Zealand	Demographic data	Total population aged greater than 60 years
New Zealand	Environmental covariates	Nightlights in nanoWatts per centimeter squared per steradian
New Zealand	Demographic data	Total population
New Zealand	Demographic data	Population density of the total population per kilometers squared
New Zealand	Health related data	Motorized travel time to a healthcare facility in minutes
New Zealand	Demographic data	Total population aged less than 5 years
New Zealand	Demographic data	Total population aged in the range of 15 to 24 years
Niue	Indicators of human development	Gridded Relative Deprivation Index
Niue	Administrative boundaries	Distance to road in kilometers
Niue	Environmental covariates	Nightlights in nanoWatts per centimeter squared per steradian
Niue	Health related data	Motorized travel time to a healthcare facility in minutes
Marshall Islands	Indicators of human development	Gridded Relative Deprivation Index
Northern Mariana Islands	Indicators of human development	Gridded Relative Deprivation Index
Northern Mariana Islands	Administrative boundaries	Distance to road in kilometers
Northern Mariana Islands	Environmental covariates	Nightlights in nanoWatts per centimeter squared per steradian
Northern Mariana Islands	Health related data	Motorized travel time to a healthcare facility in minutes
Palau	Indicators of human development	Gridded Relative Deprivation Index
Palau	Administrative boundaries	Distance to road in kilometers
Palau	Demographic data	Total population aged greater than 60 years
Palau	Environmental covariates	Nightlights in nanoWatts per centimeter squared per steradian
Palau	Demographic data	Total population
Palau	Demographic data	Population density of the total population per kilometers squared
Palau	Health related data	Motorized travel time to a healthcare facility in minutes
Palau	Demographic data	Total population aged less than 5 years
Palau	Demographic data	Total population aged in the range of 15 to 24 years
Papua New Guinea	Socio-demographic data	Distance to road in kilometers

Country name	Data source type	Indicator
Papua New Guinea	Socio-demographic data	Nightlights in nanoWatts per centimeter squared per steradian
Papua New Guinea	Socio-demographic data	Percentage of children who have received a BCG vaccination
Papua New Guinea	Socio-demographic data	Percentage of children who have received a DPT 1 vaccination
Papua New Guinea	Socio-demographic data	Percentage of children who have received a DPT 2 vaccination
Papua New Guinea	Socio-demographic data	Percentage of children who have received a DPT 3 vaccination
Papua New Guinea	Socio-demographic data	Percentage of children who have received a measles vaccination
Philippines	Socio-demographic data	Births per grid square
Philippines	Socio-demographic data	Distance to road in kilometers
Philippines	Socio-demographic data	Nightlights in nanoWatts per centimeter squared per steradian
Philippines		
Philippines	Socio-demographic data	Percentage of children experiencing underweight/malnutrition
Philippines	Socio-demographic data	Percentage of population using open defecation
Philippines	Socio-demographic data	Population density of the total population per kilometers squared
Philippines	Socio-demographic data	Relative wealth index (normalized [0-1])
Philippines	Socio-demographic data	Percentage of children experiencing stunted development
Philippines	Socio-demographic data	Total population
Philippines	Health related data	Motorized travel time to a healthcare facility in minutes
Philippines	Socio-economic data	Motorized travel time to a healthcare facility in minutes
Philippines	Socio-economic data	Total population aged less than 5 years
Philippines	Socio-economic data	Percentage of children who have received a BCG vaccination
Philippines	Socio-economic data	Percentage of children who have received a DPT 1 vaccination
Fiji	Indicators of human development	DPT 3 coverage level
Philippines	Socio-economic data	Percentage of children who have received a DPT 3 vaccination

Country name	Data source type	Indicator
Philippines	Socio-economic data	Percentage of children who have received a measles vaccination
Philippines	Socio-economic data	Percentage of population with access to clean water
Philippines	Socio-economic data	Total population aged in the range of 15 to 24 years
Republic of Korea	Administrative boundaries	Distance to road in kilometers
Republic of Korea	Demographic data	Total population aged greater than 60 years
Republic of Korea	Demographic data	Life expectency at birth
Republic of Korea	Environmental covariates	Nightlights in nanoWatts per centimeter squared per steradian
Republic of Korea	Indicators of human development	Percentage of children experiencing stunted development
Republic of Korea	Demographic data	Total population
Republic of Korea	Demographic data	Population density of the total population per kilometers squared
Republic of Korea	Health related data	Motorized travel time to a healthcare facility in minutes
Republic of Korea	Demographic data	Total population aged less than 5 years
Republic of Korea	Demographic data	Births per grid square
Republic of Korea	Demographic data	Total population aged in the range of 15 to 24 years
Republic of Korea	Administrative boundaries	
Samoa	Indicators of human development	Gridded Relative Deprivation Index
Samoa	Demographic data	Births per grid square
Samoa	Administrative boundaries	Distance to road in kilometers
Samoa	Demographic data	Total population aged greater than 60 years
Samoa	Environmental covariates	Nightlights in nanoWatts per centimeter squared per steradian
Samoa	Demographic data	Total population
Samoa	Demographic data	Population density of the total population per kilometers squared
Samoa	Health related data	Motorized travel time to a healthcare facility in minutes
Samoa	Demographic data	Total population aged less than 5 years
Samoa	Demographic data	Total population aged in the range of 15 to 24 years
Republic of Korea	Indicators of human development	Gridded Relative Deprivation Index
Singapore	Indicators of human development	Gridded Relative Deprivation Index

Country name	Data source type	Indicator
Singapore	Administrative boundaries	Distance to road in kilometers
Singapore	Demographic data	Total population aged greater than 60 years
Singapore	Environmental covariates	Nightlights in nanoWatts per centimeter squared per steradian
Singapore	Demographic data	Total population
Singapore	Demographic data	Population density of the total population per kilometers squared
Singapore	Health related data	Motorized travel time to a healthcare facility in minutes
Singapore	Demographic data	Total population aged less than 5 years
Singapore	Demographic data	Births per grid square
Solomon Islands	Indicators of human development	Gridded Relative Deprivation Index
Solomon Islands	Socio-economic data	Distance to road in kilometers
Solomon Islands	Socio-economic data	Nightlights in nanoWatts per centimeter squared per steradian
Solomon Islands	Socio-economic data	Percentage of children experiencing underweight/malnutrition
Solomon Islands	Socio-economic data	Population density of the total population per kilometers squared
Solomon Islands	Socio-economic data	Percentage of the population living below the poverty line
Solomon Islands	Socio-economic data	Percentage of children experiencing stunted development
Solomon Islands	Socio-economic data	Total population
Solomon Islands	Socio-economic data	Motorized travel time to a healthcare facility in minutes
Solomon Islands	Socio-economic data	Total population aged less than 5 years
French Polynesia	Indicators of human development	DPT 3 coverage level
Solomon Islands	Socio-economic data	Total population aged in the range of 15 to 24 years
Solomon Islands	Administrative boundaries	
Tokelau	Indicators of human development	Gridded Relative Deprivation Index
Tokelau	Administrative boundaries	Distance to road in kilometers
Tokelau	Environmental covariates	Nightlights in nanoWatts per centimeter squared per steradian
Tokelau	Health related data	Motorized travel time to a healthcare facility in minutes
Tonga	Socio-economic data	Births per grid square
Tonga	Socio-economic data	Distance to road in kilometers

Country name	Data source type	Indicator
Tonga	Socio-economic data	Total population aged greater than 60 years
Tonga	Socio-economic data	International wealth index
Tonga	Socio-economic data	Literacy rate
Tonga	Socio-economic data	Mortality rate of children under 5 years
Tonga	Socio-economic data	Nightlights in nanoWatts per centimeter squared per steradian
Tonga	Socio-economic data	Percentage of children experiencing underweight/malnutrition
Tonga	Socio-economic data	Population density of the total population per kilometers squared
Tonga	Socio-economic data	Percentage of children experiencing stunted development
Tonga	Socio-economic data	Total population
Tonga	Socio-economic data	Motorized travel time to a healthcare facility in minutes
Tonga	Socio-economic data	Total population aged less than 5 years
Guam	Indicators of human development	DPT 3 coverage level
Tonga		Estimated vulnerability index (normalized [0-1])
Tonga	Socio-economic data	Total population aged in the range of 15 to 24 years
Tonga	Administrative boundaries	
Tuvalu	Indicators of human development	Gridded Relative Deprivation Index
Tuvalu	Demographic data	Births per grid square
Tuvalu	Administrative boundaries	Distance to road in kilometers
Tuvalu	Demographic data	Total population aged greater than 60 years
Tuvalu	Indicators of human development	International wealth index
Tuvalu	Indicators of human development	Mortality rate of children under 5 years
Tuvalu	Environmental covariates	Nightlights in nanoWatts per centimeter squared per steradian
Tuvalu	Indicators of human development	Percentage of children experiencing stunted development
Tuvalu	Demographic data	Total population
Tuvalu	Demographic data	Population density of the total population per kilometers squared
Tuvalu	Health related data	Motorized travel time to a healthcare facility in minutes

Country name	Data source type	Indicator
Tuvalu	Demographic data	Total population aged less than 5 years
Tuvalu	Demographic data	Total population aged in the range of 15 to 24 years
Tuvalu	Administrative boundaries	
Vanuatu	Socio-economic data	Births per grid square
Vanuatu	Socio-economic data	Distance to road in kilometers
Vanuatu	Socio-economic data	Total population aged greater than 60 years
Vanuatu	Socio-economic data	International wealth index
Vanuatu	Demographic data	Life expectancy at birth
Vanuatu	Socio-economic data	Literacy rate
Vanuatu	Socio-economic data	Mortality rate of children under 5 years
Vanuatu	Socio-economic data	Nightlights in nanoWatts per centimeter squared per steradian
Vanuatu	Socio-economic data	Percentage of children experiencing underweight/malnutrition
Vanuatu	Socio-economic data	Population density of the total population per kilometers squared
Vanuatu	Socio-economic data	Percentage of children experiencing stunted development
Vanuatu	Socio-economic data	Total population
Vanuatu	Socio-economic data	Motorized travel time to a healthcare facility in minutes
Vanuatu	Socio-economic data	Total population aged less than 5 years
Japan	Indicators of human development	DPT 3 coverage level
Vanuatu		Estimated vulnerability index (normalized [0-1])
Vanuatu	Socio-economic data	Total population aged in the range of 15 to 24 years
Vanuatu	Administrative boundaries	
Viet Nam	Socio-economic data	Births per grid square
Viet Nam	Socio-economic data	Distance to road in kilometers
Viet Nam	Socio-economic data	Total population aged greater than 60 years
Viet Nam		Regions with both a 75 percentile poverty rate and a 75 percent vaccination rate
Viet Nam	Socio-economic data	International wealth index

Country name	Data source type	Indicator
Viet Nam	Demographic data	Life expectancy at birth
Viet Nam	Demographic data	Life expectancy at birth
Viet Nam	Socio-economic data	Mortality rate of children under 5 years
Viet Nam	Socio-economic data	Nightlights in nanoWatts per centimeter squared per steradian
Viet Nam	Socio-economic data	Percentage of children experiencing underweight/malnutrition
Viet Nam	Socio-economic data	Percentage of population using open defecation
Viet Nam	Socio-economic data	Population density of the total population per kilometers squared
Viet Nam	Socio-economic data	Relative wealth index (normalized [0-1])
Viet Nam	Socio-economic data	Percentage of children experiencing stunted development
Viet Nam	Socio-economic data	Total population
Viet Nam	Socio-economic data	Motorized travel time to a healthcare facility in minutes
Viet Nam	Socio-economic data	Total population aged less than 5 years
Kiribati	Indicators of human development	DPT 3 coverage level
Viet Nam		Estimated vulnerability index (normalized [0-1])
Viet Nam	Socio-economic data	Percentage of population with access to clean water
Viet Nam	Socio-economic data	Total population aged in the range of 15 to 24 years
Viet Nam	Administrative boundaries	
Wallis and Futuna	Indicators of human development	Gridded Relative Deprivation Index
Wallis and Futuna	Administrative boundaries	Distance to road in kilometers
Wallis and Futuna	Demographic data	Total population aged greater than 60 years
Wallis and Futuna	Indicators of human development	Literacy rate
Wallis and Futuna	Indicators of human development	Literacy rate of women
Wallis and Futuna	Environmental covariates	Nightlights in nanoWatts per centimeter squared per steradian
Wallis and Futuna	Health related data	Motorized travel time to a healthcare facility in minutes
Wallis and Futuna	Administrative boundaries	
		Life expectancy at birth

Country name	Data source type	Indicator
Lao People's Democratic Republic (the)	Indicators of human development	DPT 3 coverage level
Malaysia	Indicators of human development	DPT 3 coverage level
Marshall Islands	Indicators of human development	DPT 3 coverage level
Micronesia (Federated States of)	Indicators of human development	DPT 3 coverage level
Mongolia	Indicators of human development	DPT 3 coverage level
Nauru	Indicators of human development	DPT 3 coverage level
New Caledonia	Indicators of human development	DPT 3 coverage level
New Zealand	Indicators of human development	DPT 3 coverage level
Niue	Indicators of human development	DPT 3 coverage level
Northern Mariana Islands	Indicators of human development	DPT 3 coverage level
Palau	Indicators of human development	DPT 3 coverage level
Papua New Guinea	Indicators of human development	DPT 3 coverage level
Philippines	Indicators of human development	DPT 3 coverage level
Republic of Korea	Indicators of human development	DPT 3 coverage level
Samoa	Indicators of human development	DPT 3 coverage level
Singapore	Indicators of human development	DPT 3 coverage level
Solomon Islands	Indicators of human development	DPT 3 coverage level
Tokelau	Indicators of human development	DPT 3 coverage level
Tonga	Indicators of human development	DPT 3 coverage level
Tuvalu	Indicators of human development	DPT 3 coverage level
Vanuatu	Indicators of human development	DPT 3 coverage level

Country name	Data source type	Indicator
Viet Nam	Indicators of human development	DPT 3 coverage level
Wallis and Futuna	Indicators of human development	DPT 3 coverage level

Country name	Data source type	Source	Year	Resolution
American Samoa	Indicators of human development	Earth Science Data Systems Program	2020	1km
American Samoa	Administrative boundaries	WorldPop	2018	100m
American Samoa	Demographic data	Facebook	2020	100m
American Samoa	Environmental covariates	Earth Observation Group	2024	500m
American Samoa	Demographic data	Facebook	2020	100m
American Samoa	Demographic data	Facebook	2020	100m
American Samoa	Health related data	Malaria Atlas	2019	1 km
American Samoa	Demographic data	Facebook	2020	100m
American Samoa	Demographic data	Facebook	2020	100m
Australia	Indicators of human development	Earth Science Data Systems Program	2020	1km
Australia	Administrative boundaries	WorldPop	2018	100m
Australia	Demographic data	Facebook	2019	100m
Australia	Indicators of human development	Australian Child Death Statistics	2021	admin 1
Australia	Environmental covariates	Earth Observation Group	2024	500m
Australia	Indicators of human development	IHME	2019	100m
Australia	Demographic data	WorldPop	2020	100m
Australia	Demographic data	Facebook	2020	100m
Australia	Health related data	Malaria Atlas	2019	1 km
Australia	Demographic data	Facebook	2019	100m
Brunei Darussalam	Indicators of human development	Earth Science Data Systems Program	2020	1km
Brunei Darussalam	Demographic data	WorldPop	2018	1km
Brunei Darussalam	Demographic data	Facebook	2020	100m
Brunei Darussalam	Administrative boundaries	WorldPop	2016	100m
Brunei Darussalam	Demographic data	Facebook	2020	100m
Brunei Darussalam	Indicators of human development	IHME	2017	100m

Country name	Data source type	Source	Year	Resolution
Brunei Darussalam	Environmental covariates	Earth Observation Group	2024	500m
Brunei Darussalam	Demographic data	Facebook	2020	100m
Brunei Darussalam	Demographic data	Facebook	2020	100m
Brunei Darussalam	Health related data	Malaria Atlas	2019	1 km
Brunei Darussalam	Demographic data	Facebook	2020	100m
Australia	Demographic data	Facebook	2019	100m
Cambodia	Indicators of human development	WorldPop	2016	100m
Cambodia	Indicators of human development	Earth Observation Group	2024	500m
Cambodia	Indicators of human development	DHS	2021	5x5 km
China	Indicators of human development	Earth Science Data Systems Program	2020	1km
China	Demographic data	WorldPop	2018	1km
China	Administrative boundaries	WorldPop	2016	100m
China	Indicators of human development	Global Data Lab	2018	admin 1
China	Demographic data	The Seventh National Population Census	2020	admin 1
China	Indicators of human development	National Bureau of Statistics of China	2024	admin 1
China	Indicators of human development	IHME	2017	100m
China	Indicators of human development	Global Data Lab, IHME	2017	100m
China	Environmental covariates	Earth Observation Group	2024	500m
China	Indicators of human development	IHME	2019	100m
China	Indicators of human development	IHME	2019	100m
China	Demographic data	Facebook	2020	100m
China	Demographic data	Facebook	2020	100m
China	Health related data	Malaria Atlas	2019	1 km
Cook Islands	Indicators of human development	Earth Science Data Systems Program	2020	1km
Cook Islands	Administrative boundaries	WorldPop	2016	100m

Country name	Data source type	Source	Year	Resolution
Cook Islands	Environmental covariates	Earth Observation Group	2024	500m
Cook Islands	Health related data	Malaria Atlas	2019	1 km
Fiji	Indicators of human development	Earth Science Data Systems Program	2020	1km
Fiji	Administrative boundaries	WorldPop	2016	100m
Fiji	Demographic data	Facebook	2020	100m
Fiji	Indicators of human development	Global Data Lab	2023	admin 2
Fiji	Indicators of human development	Global Data Lab	2023	admin 2
Fiji	Environmental covariates	Earth Observation Group	2024	500m
Fiji	Demographic data	Facebook	2020	100m
Fiji	Demographic data	Facebook	2020	100m
Fiji	Health related data	Malaria Atlas	2019	1 km
Fiji	Demographic data	Facebook	2020	100m
Fiji	Demographic data	Facebook	2019	100m
French Polynesia	Indicators of human development	Earth Science Data Systems Program	2020	1km
French Polynesia	Administrative boundaries	WorldPop	2016	100m
French Polynesia	Environmental covariates	Earth Observation Group	2024	500m
French Polynesia	Health related data	Malaria Atlas	2019	1 km
Guam	Indicators of human development	Earth Science Data Systems Program	2020	1km
Guam	Demographic data	Facebook	2020	100m
Guam	Environmental covariates	Earth Observation Group	2024	500m
Guam	Demographic data	Facebook	2020	100m
Guam	Demographic data	Facebook	2020	100m
Guam	Health related data	Malaria Atlas	2019	1 km
Guam	Demographic data	Facebook	2020	100m
Guam	Administrative boundaries	WorldPop	2016	100m

Country name	Data source type	Source	Year	Resolution
Guam	Demographic data	Facebook	2020	100m
China, Hong Kong SAR	Indicators of human development	Earth Science Data Systems Program	2020	1km
China, Hong Kong SAR	Administrative boundaries	WorldPop	2016	100m
China, Hong Kong SAR	Demographic data	World Bank	2021	admin 1
China, Macao SAR	Demographic data	World Bank	2021	admin 1
China, Hong Kong SAR	Environmental covariates	Earth Observation Group	2024	500m
China, Hong Kong SAR	Health related data	Malaria Atlas	2019	1 km
Japan	Indicators of human development	Earth Science Data Systems Program	2020	1km
Japan	Administrative boundaries	WorldPop	2016	100m
Japan	Demographic data	Facebook	2019	100m
Japan	Environmental covariates	Earth Observation Group	2024	500m
Japan	Demographic data	Facebook	2020	100m
Japan	Demographic data	Facebook	2020	100m
Japan	Health related data	Malaria Atlas	2019	1 km
Japan	Demographic data	Facebook	2019	100m
Japan	Demographic data	WorldPop	2018	1km
Japan	Demographic data	Facebook	2019	100m
Kiribati	Indicators of human development	Earth Science Data Systems Program	2020	1km
Kiribati	Demographic data	Facebook	2019	100m
Kiribati	Environmental covariates	Earth Observation Group	2024	500m
Kiribati	Demographic data	Facebook	2018	100m
Kiribati	Demographic data	Facebook	2019	100m
Kiribati	Health related data	Malaria Atlas	2020	1 km
Kiribati	Demographic data	Facebook	2019	100m
Kiribati	Administrative boundaries	WorldPop	2016	100m
Kiribati	Demographic data	Facebook	2019	100m
Lao People's Democratic Republic (the)	Indicators of human development	WorldPop	2016	100m

Country name	Data source type	Source	Year	Resolution
Lao People's Democratic Republic (the)	Indicators of human development	Earth Observation Group	2024	500m
Lao People's Democratic Republic (the)	Indicators of human development	MICS	2017	Region level
China, Macao SAR	Indicators of human development	Earth Science Data Systems Program	2020	1km
China, Macao SAR	Administrative boundaries	WorldPop	2016	100m
China, Macao SAR	Environmental covariates	Earth Observation Group	2024	500m
China, Macao SAR	Demographic data	Facebook	2020	100m
China, Macao SAR	Demographic data	Facebook	2020	100m
China, Macao SAR	Health related data	Malaria Atlas	2019	1 km
Malaysia	Indicators of human development	WorldPop	2016	100m
Malaysia	Indicators of human development	Earth Observation Group	2024	500m
China, Hong Kong SAR	Indicators of human development	WHO/UNICEF Electronic Joint Reporting Form on Immunization		
Marshall Islands	Indicators of human development	Earth Science Data Systems Program	2020	1km
Marshall Islands	Administrative boundaries	WorldPop	2016	100m
Marshall Islands	Environmental covariates	Earth Observation Group	2024	500m
Marshall Islands	Health related data	Malaria Atlas	2019	1 km
Micronesia (Federated States of)	Indicators of human development	Earth Science Data Systems Program	2020	1km
Micronesia (Federated States of)	Administrative boundaries	WorldPop	2016	100m
Micronesia (Federated States of)	Demographic data	Facebook	2020	100m
Micronesia (Federated States of)	Environmental covariates	Earth Observation Group	2024	500m
Micronesia (Federated States of)	Demographic data	Facebook	2020	100m
Micronesia (Federated States of)	Demographic data	Facebook	2020	100m
Micronesia (Federated States of)	Health related data	Malaria Atlas	2019	1 km

Country name	Data source type	Source	Year	Resolution
Micronesia (Federated States of)	Demographic data	Facebook	2020	100m
Micronesia (Federated States of)	Demographic data	Facebook	2020	100m
Mongolia	Indicators of human development	WorldPop	2016	100m
Mongolia	Indicators of human development	Earth Observation Group	2024	500m
Mongolia	Indicators of human development	MICS	2018	Region level
Nauru	Indicators of human development	Earth Science Data Systems Program	2020	1km
Nauru	Administrative boundaries	WorldPop	2016	100m
Nauru	Demographic data	Facebook	2020	100m
Nauru	Environmental covariates	Earth Observation Group	2024	500m
Nauru	Demographic data	Facebook	2020	100m
Nauru	Demographic data	Facebook	2020	100m
Nauru	Health related data	Malaria Atlas	2019	1 km
Nauru	Demographic data	Facebook	2020	100m
Nauru	Demographic data	Facebook	2020	100m
New Caledonia	Indicators of human development	Earth Science Data Systems Program	2020	1km
New Caledonia	Administrative boundaries	WorldPop	2016	100m
New Caledonia	Demographic data	Facebook	2019	100m
New Caledonia	Environmental covariates	Earth Observation Group	2024	500m
New Caledonia	Demographic data	Facebook	2019	100m
New Caledonia	Demographic data	Facebook	2019	100m
New Caledonia	Health related data	Malaria Atlas	2019	1 km
New Caledonia	Demographic data	Facebook	2019	100m
New Caledonia	Demographic data	Facebook	2019	100m
New Zealand	Indicators of human development	Earth Science Data Systems Program	2020	1km
New Zealand	Administrative boundaries	WorldPop	2016	100m

Country name	Data source type	Source	Year	Resolution
New Zealand	Demographic data	Facebook	2019	100m
New Zealand	Environmental covariates	Earth Observation Group	2024	500m
New Zealand	Demographic data	Facebook	2019	100m
New Zealand	Demographic data	Facebook	2019	100m
New Zealand	Health related data	Malaria Atlas	2019	1 km
New Zealand	Demographic data	Facebook	2019	100m
New Zealand	Demographic data	Facebook	2019	100m
Niue	Indicators of human development	Earth Science Data Systems Program	2020	1km
Niue	Administrative boundaries	WorldPop	2016	100m
Niue	Environmental covariates	Earth Observation Group	2024	500m
Niue	Health related data	Malaria Atlas	2019	1 km
Marshall Islands	Indicators of human development	Earth Science Data Systems Program	2020	1km
Northern Mariana Islands	Indicators of human development	Earth Science Data Systems Program	2020	1km
Northern Mariana Islands	Administrative boundaries	WorldPop	2016	100m
Northern Mariana Islands	Environmental covariates	Earth Observation Group	2024	500m
Northern Mariana Islands	Health related data	Malaria Atlas	2019	1 km
Palau	Indicators of human development	Earth Science Data Systems Program	2020	1km
Palau	Administrative boundaries	WorldPop	2016	100m
Palau	Demographic data	Facebook	2020	100m
Palau	Environmental covariates	Earth Observation Group	2024	500m
Palau	Demographic data	Facebook	2020	100m
Palau	Demographic data	Facebook	2020	100m
Palau	Health related data	Malaria Atlas	2019	1 km
Palau	Demographic data	Facebook	2020	100m
Palau	Demographic data	Facebook	2020	100m
Papua New Guinea	Socio-demographic data	WorldPop	2016	100m

Country name	Data source type	Source	Year	Resolution
Papua New Guinea	Socio-demographic data	Earth Observation Group	2024	500m
Papua New Guinea	Socio-demographic data	Global Data Lab	2021	
Papua New Guinea	Socio-demographic data	Global Data Lab	2021	
Papua New Guinea	Socio-demographic data	Global Data Lab	2021	
Papua New Guinea	Socio-demographic data	Global Data Lab	2021	
Papua New Guinea	Socio-demographic data	Global Data Lab	2021	
Philippines	Socio-demographic data	WorldPop	2018	
Philippines	Socio-demographic data	WorldPop	2016	100m
Philippines	Socio-demographic data	Earth Observation Group	2024	500m
Philippines		Earth Observation Group	2024	500m
Philippines	Socio-demographic data	IHME	2019	
Philippines	Socio-demographic data	DHS	2022	
Philippines	Socio-demographic data	Facebook	calculated from the above data of year 2020	
Philippines	Socio-demographic data	Facebook	2021	
Philippines	Socio-demographic data	IHME	2019	
Philippines	Socio-demographic data	Facebook	2020	
Philippines	Health related data	Malaria Atlas	2019	1 km
Philippines	Socio-economic data	Malaria Atlas	2019	
Philippines	Socio-economic data	Facebook	2020	
Philippines	Socio-economic data	DHS	2022	
Philippines	Socio-economic data	DHS	2022	
Fiji	Indicators of human development	WHO/UNICEF Electronic Joint Reporting Form on Immunization	2020	
Philippines	Socio-economic data	DHS	2022	

Country name	Data source type	Source	Year	Resolution
Philippines	Socio-economic data	DHS	2022	
Philippines	Socio-economic data	DHS	2022	
Philippines	Socio-economic data	Facebook		2020
Republic of Korea	Administrative boundaries	WorldPop	2016	100m
Republic of Korea	Demographic data	Facebook	2018	100m
Republic of Korea	Demographic data	KOSIS, Vital Statistics Division, Social Statistics Bureau, Republic of Korea	2020	
Republic of Korea	Environmental covariates	Earth Observation Group	2024	500m
Republic of Korea	Indicators of human development	IHME	2010	
Republic of Korea	Demographic data	Facebook	2018	100m
Republic of Korea	Demographic data	Facebook	2018	100m
Republic of Korea	Health related data	Malaria Atlas	2019	1 km
Republic of Korea	Demographic data	Facebook	2018	100m
Republic of Korea	Demographic data	WorldPop	2018	1km
Republic of Korea	Demographic data	Facebook	2018	100m
Republic of Korea	Administrative boundaries	HDX		
Samoa	Indicators of human development	Earth Science Data Systems Program	2020	1km
Samoa	Demographic data	-		-
Samoa	Administrative boundaries	WorldPop	2016	100m
Samoa	Demographic data	Facebook	2018	100m
Samoa	Environmental covariates	Earth Observation Group	2024	500m
Samoa	Demographic data	Facebook	2018	100m
Samoa	Demographic data	Facebook	2018	100m
Samoa	Health related data	Malaria Atlas	2019	1 km
Samoa	Demographic data	Facebook	2018	100m
Samoa	Demographic data	Facebook	2018	100m
Republic of Korea	Indicators of human development	Earth Science Data Systems Program	2020	1km
Singapore	Indicators of human development	Earth Science Data Systems Program	2020	1km

Country name	Data source type	Source	Year	Resolution
Singapore	Administrative boundaries	WorldPop	2016	100m
Singapore	Demographic data	Facebook	2020	100m
Singapore	Environmental covariates	Earth Observation Group	2024	500m
Singapore	Demographic data	Facebook	2020	100m
Singapore	Demographic data	Facebook	2020	100m
Singapore	Health related data	Malaria Atlas	2019	1 km
Singapore	Demographic data	Facebook	2020	100m
Singapore	Demographic data	WorldPop	2018	1km
Solomon Islands	Indicators of human development	Earth Science Data Systems Program	2020	1km
Solomon Islands	Socio-economic data	WorldPop	2016	100m
Solomon Islands	Socio-economic data	Earth Observation Group	2024	500m
Solomon Islands	Socio-economic data	IHME	2019	
Solomon Islands	Socio-economic data	Facebook	2019	
Solomon Islands	Socio-economic data	Solomon Islands Government website	2017	
Solomon Islands	Socio-economic data	IHME	2019	
Solomon Islands	Socio-economic data	Facebook	2019	
Solomon Islands	Socio-economic data	Malaria Atlas	2019	
Solomon Islands	Socio-economic data	Facebook	2019	
French Polynesia	Indicators of human development	WHO/UNICEF Electronic Joint Reporting Form on Immunization		
Solomon Islands	Socio-economic data	Facebook	2019	
Solomon Islands	Administrative boundaries	GADM		
Tokelau	Indicators of human development	Earth Science Data Systems Program	2020	1km
Tokelau	Administrative boundaries	WorldPop	2016	100m
Tokelau	Environmental covariates	Earth Observation Group	2024	500m
Tokelau	Health related data	Malaria Atlas	2019	1 km
Tonga	Socio-economic data	WorldPop	2018	
Tonga	Socio-economic data	WorldPop	2016	100m

Country name	Data source type	Source	Year	Resolution
Tonga	Socio-economic data	Facebook	2020	
Tonga	Socio-economic data	Global Data Lab	2019	
Tonga	Socio-economic data	Global Data Lab	2019	
Tonga	Socio-economic data	IHME	2017	
Tonga	Socio-economic data	Earth Observation Group	2024	500m
Tonga	Socio-economic data	IHME	2019	
Tonga	Socio-economic data	Facebook	calculated from the above data of year 2020	
Tonga	Socio-economic data	IHME	2019	
Tonga	Socio-economic data	Facebook	2020	
Tonga	Socio-economic data	Malaria Atlas	2019	
Tonga	Socio-economic data	Facebook	2020	
Guam	Indicators of human development	WHO/UNICEF Electronic Joint Reporting Form on Immunization		
Tonga		Modelled by Reaching the Unreached: Map explorer WPR project	for details go to the help page.	
Tonga	Socio-economic data	Facebook	2020	
Tonga	Administrative boundaries	GADM		
Tuvalu	Indicators of human development	Earth Science Data Systems Program	2020	1km
Tuvalu	Demographic data	-	-	
Tuvalu	Administrative boundaries	WorldPop	2016	100m
Tuvalu	Demographic data	Facebook	2020	100m
Tuvalu	Indicators of human development	Global Data Lab	2023 estimate	admin 1
Tuvalu	Indicators of human development	Global Data Lab	2023	admin 1
Tuvalu	Environmental covariates	Earth Observation Group	2024	500m
Tuvalu	Indicators of human development	IHME	2019	
Tuvalu	Demographic data	Facebook	-	
Tuvalu	Demographic data	Facebook	-	
Tuvalu	Health related data	Malaria Atlas	2019	1 km

Country name	Data source type	Source	Year	Resolution
Tuvalu	Demographic data	Facebook	2020	100m
Tuvalu	Demographic data	Facebook	2020	100m
Tuvalu	Administrative boundaries	GADM		
Vanuatu	Socio-economic data	WorldPop	2018	
Vanuatu	Socio-economic data	WorldPop	2016	100m
Vanuatu	Socio-economic data	Facebook	2019	
Vanuatu	Socio-economic data	Global Data Lab	2007	
Vanuatu	Demographic data	Statistical Yearbook of Viet Nam 2022 (preliminary da	2022	
Vanuatu	Socio-economic data	Global Data Lab	2007	
Vanuatu	Socio-economic data	IHME	2017	
Vanuatu	Socio-economic data	Earth Observation Group	2024	500m
Vanuatu	Socio-economic data	IHME	2019	
Vanuatu	Socio-economic data	Facebook	2019	
Vanuatu	Socio-economic data	IHME	2019	
Vanuatu	Socio-economic data	Facebook	2019	
Vanuatu	Socio-economic data	Malaria Atlas	2019	
Vanuatu	Socio-economic data	Facebook	2019	
Japan	Indicators of human development	WHO/UNICEF Electronic Joint Reporting Form on Immunization	2020	
Vanuatu		Modelled by Reaching the Unreached: Map explorer WPR project	for details go to the help page.	
Vanuatu	Socio-economic data	Facebook	2019	
Vanuatu	Administrative boundaries	GADM		
Viet Nam	Socio-economic data	WorldPop	2018	
Viet Nam	Socio-economic data	WorldPop	2016	100m
Viet Nam	Socio-economic data	Facebook	2020	
Viet Nam		Modelled by Reaching the Unreached: Map explorer WPR project	for details go to the help page.	
Viet Nam	Socio-economic data	Global Data Lab	2021	

Country name	Data source type	Source	Year	Resolution
Viet Nam	Demographic data	Modelled by Reaching the Unreached: Map explorer WPR project	for details go to the help page.	
Viet Nam	Demographic data	-	-	
Viet Nam	Socio-economic data	IHME	2017	
Viet Nam	Socio-economic data	Earth Observation Group	2024	500m
Viet Nam	Socio-economic data	IHME	2019	
Viet Nam	Socio-economic data	Global Data Lab	2005	
Viet Nam	Socio-economic data	Facebook	2020	
Viet Nam	Socio-economic data	Facebook	2021	
Viet Nam	Socio-economic data	IHME	2019	
Viet Nam	Socio-economic data	Facebook	2020	
Viet Nam	Socio-economic data	Malaria Atlas	2019	
Viet Nam	Socio-economic data	Facebook	2020	
Kiribati	Indicators of human development	WHO/UNICEF Electronic Joint Reporting Form on Immunization	2022	
Viet Nam		Modelled by Reaching the Unreached: Map explorer WPR project	for details go to the help page.	
Viet Nam	Socio-economic data	Global Data Lab	2005	
Viet Nam	Socio-economic data	Facebook	2020	
Viet Nam	Administrative boundaries	GADM		
Wallis and Futuna	Indicators of human development	Earth Science Data Systems Program	2020	1km
Wallis and Futuna	Administrative boundaries	WorldPop	2016	100m
Wallis and Futuna	Demographic data	Facebook	-	
Wallis and Futuna	Indicators of human development	Global Data Lab, IHME	-	
Wallis and Futuna	Indicators of human development	Global Data Lab, IHME	-	
Wallis and Futuna	Environmental covariates	Earth Observation Group	2024	500m
Wallis and Futuna	Health related data	Malaria Atlas	2019	1 km
Wallis and Futuna	Administrative boundaries	GADM		
		Global Data Lab	2021	

Country name	Data source type	Source	Year	Resolution
Lao People's Democratic Republic (the)	Indicators of human development	WHO/UNICEF Electronic Joint Reporting Form on Immunization	2022	
Malaysia	Indicators of human development	WHO/UNICEF Electronic Joint Reporting Form on Immunization	2022	
Marshall Islands	Indicators of human development	WHO/UNICEF Electronic Joint Reporting Form on Immunization	2019	
Micronesia (Federated States of)	Indicators of human development	WHO/UNICEF Electronic Joint Reporting Form on Immunization	2022	
Mongolia	Indicators of human development	WHO/UNICEF Electronic Joint Reporting Form on Immunization	2022	
Nauru	Indicators of human development	WHO/UNICEF Electronic Joint Reporting Form on Immunization		
New Caledonia	Indicators of human development	WHO/UNICEF Electronic Joint Reporting Form on Immunization		
New Zealand	Indicators of human development	WHO/UNICEF Electronic Joint Reporting Form on Immunization	2022	
Niue	Indicators of human development	WHO/UNICEF Electronic Joint Reporting Form on Immunization		
Northern Mariana Islands	Indicators of human development	WHO/UNICEF Electronic Joint Reporting Form on Immunization		
Palau	Indicators of human development	WHO/UNICEF Electronic Joint Reporting Form on Immunization		
Papua New Guinea	Indicators of human development	WHO/UNICEF Electronic Joint Reporting Form on Immunization	2022	
Philippines	Indicators of human development	WHO/UNICEF Electronic Joint Reporting Form on Immunization	2022	-
Republic of Korea	Indicators of human development	WHO/UNICEF Electronic Joint Reporting Form on Immunization	2021	
Samoa	Indicators of human development	WHO/UNICEF Electronic Joint Reporting Form on Immunization	2022	
Singapore	Indicators of human development	WHO/UNICEF Electronic Joint Reporting Form on Immunization		
Solomon Islands	Indicators of human development	WHO/UNICEF Electronic Joint Reporting Form on Immunization	2021	
Tokelau	Indicators of human development	WHO/UNICEF Electronic Joint Reporting Form on Immunization		
Tonga	Indicators of human development	WHO/UNICEF Electronic Joint Reporting Form on Immunization	2018	
Tuvalu	Indicators of human development	WHO/UNICEF Electronic Joint Reporting Form on Immunization	2022	
Vanuatu	Indicators of human development	WHO/UNICEF Electronic Joint Reporting Form on Immunization	2022	

Country name	Data source type	Source	Year	Resolution
Viet Nam	Indicators of human development	WHO/UNICEF Electronic Joint Reporting Form on Immunization	2020	
Wallis and Futuna	Indicators of human development	WHO/UNICEF Electronic Joint Reporting Form on Immunization		

Country name	Data source type	Unit
American Samoa	Indicators of human development	Index
American Samoa	Administrative boundaries	km
American Samoa	Demographic data	Count
American Samoa	Environmental covariates	nW/cm2/sr
American Samoa	Demographic data	Count
American Samoa	Demographic data	Count/Area
American Samoa	Health related data	minutes
American Samoa	Demographic data	Count
American Samoa	Demographic data	Count
Australia	Indicators of human development	Index
Australia	Administrative boundaries	km
Australia	Demographic data	Count
Australia	Indicators of human development	%
Australia	Environmental covariates	nW/cm2/sr
Australia	Indicators of human development	Percent
Australia	Demographic data	Count
Australia	Demographic data	Count/Area
Australia	Health related data	minutes
Australia	Demographic data	Count
Brunei Darussalam	Indicators of human development	Index
Brunei Darussalam	Demographic data	count
Brunei Darussalam	Demographic data	count
Brunei Darussalam	Administrative boundaries	km
Brunei Darussalam	Demographic data	count
Brunei Darussalam	Indicators of human development	%

Country name	Data source type	Unit
Brunei Darussalam	Environmental covariates	nW/cm2/sr
Brunei Darussalam	Demographic data	count
Brunei Darussalam	Demographic data	Count/Area
Brunei Darussalam	Health related data	minutes
Brunei Darussalam	Demographic data	count
Australia	Demographic data	Count
Cambodia	Indicators of human development	km
Cambodia	Indicators of human development	nW/cm2/sr
Cambodia	Indicators of human development	%
China	Indicators of human development	Index
China	Demographic data	count
China	Administrative boundaries	km
China	Indicators of human development	wealth index that runs from 0 (no assets) to 100 (all assets)
China	Demographic data	years
China	Indicators of human development	%
China	Indicators of human development	%
China	Indicators of human development	%
China	Environmental covariates	nW/cm2/sr
China	Indicators of human development	Percent
China	Indicators of human development	Percent
China	Demographic data	count
China	Demographic data	Count/Area
China	Health related data	minutes
Cook Islands	Indicators of human development	Index
Cook Islands	Administrative boundaries	km

Country name	Data source type	Unit
Cook Islands	Environmental covariates	nW/cm2/sr
Cook Islands	Health related data	minutes
Fiji	Indicators of human development	Index
Fiji	Administrative boundaries	km
Fiji	Demographic data	count
Fiji	Indicators of human development	wealth index that runs from 0 (no assets) to 100 (all assets)
Fiji	Indicators of human development	%
Fiji	Environmental covariates	nW/cm2/sr
Fiji	Demographic data	count
Fiji	Demographic data	Count/Area
Fiji	Health related data	minutes
Fiji	Demographic data	count
Fiji	Demographic data	count
French Polynesia	Indicators of human development	Index
French Polynesia	Administrative boundaries	km
French Polynesia	Environmental covariates	nW/cm2/sr
French Polynesia	Health related data	minutes
Guam	Indicators of human development	Index
Guam	Demographic data	Count
Guam	Environmental covariates	nW/cm2/sr
Guam	Demographic data	Count
Guam	Demographic data	Count/Area
Guam	Health related data	minutes
Guam	Demographic data	Count
Guam	Administrative boundaries	km

Country name	Data source type	Unit
Guam	Demographic data	Count
China, Hong Kong SAR	Indicators of human development	Index
China, Hong Kong SAR	Administrative boundaries	km
China, Hong Kong SAR	Demographic data	years
China, Macao SAR	Demographic data	years
China, Hong Kong SAR	Environmental covariates	nW/cm2/sr
China, Hong Kong SAR	Health related data	minutes
Japan	Indicators of human development	Index
Japan	Administrative boundaries	km
Japan	Demographic data	count
Japan	Environmental covariates	nW/cm2/sr
Japan	Demographic data	count
Japan	Demographic data	count/Area
Japan	Health related data	minutes
Japan	Demographic data	count
Japan	Demographic data	count
Japan	Demographic data	count
Kiribati	Indicators of human development	Index
Kiribati	Demographic data	Count
Kiribati	Environmental covariates	nW/cm2/sr
Kiribati	Demographic data	Count
Kiribati	Demographic data	Count/Area
Kiribati	Health related data	minutes
Kiribati	Demographic data	Count
Kiribati	Administrative boundaries	km
Kiribati	Demographic data	Count
Lao People's Democratic Republic (the)	Indicators of human development	km

Country name	Data source type	Unit
Lao People's Democratic Republic (the)	Indicators of human development	nW/cm2/sr
Lao People's Democratic Republic (the)	Indicators of human development	%
China, Macao SAR	Indicators of human development	Index
China, Macao SAR	Administrative boundaries	km
China, Macao SAR	Environmental covariates	nW/cm2/sr
China, Macao SAR	Demographic data	count
China, Macao SAR	Demographic data	count/area
China, Macao SAR	Health related data	minutes
Malaysia	Indicators of human development	km
Malaysia	Indicators of human development	nW/cm2/sr
China, Hong Kong SAR	Indicators of human development	
Marshall Islands	Indicators of human development	Index
Marshall Islands	Administrative boundaries	km
Marshall Islands	Environmental covariates	nW/cm2/sr
Marshall Islands	Health related data	minutes
Micronesia (Federated States of)	Indicators of human development	Index
Micronesia (Federated States of)	Administrative boundaries	km
Micronesia (Federated States of)	Demographic data	Count
Micronesia (Federated States of)	Environmental covariates	nW/cm2/sr
Micronesia (Federated States of)	Demographic data	Count
Micronesia (Federated States of)	Demographic data	Count/Area
Micronesia (Federated States of)	Health related data	minutes

Country name	Data source type	Unit
Micronesia (Federated States of)	Demographic data	Count
Micronesia (Federated States of)	Demographic data	Count
Mongolia	Indicators of human development	km
Mongolia	Indicators of human development	nW/cm2/sr
Mongolia	Indicators of human development	%
Nauru	Indicators of human development	Index
Nauru	Administrative boundaries	km
Nauru	Demographic data	Count
Nauru	Environmental covariates	nW/cm2/sr
Nauru	Demographic data	Count
Nauru	Demographic data	Count/Area
Nauru	Health related data	minutes
Nauru	Demographic data	Count
Nauru	Demographic data	Count
New Caledonia	Indicators of human development	Index
New Caledonia	Administrative boundaries	km
New Caledonia	Demographic data	Count
New Caledonia	Environmental covariates	nW/cm2/sr
New Caledonia	Demographic data	Count
New Caledonia	Demographic data	Count/Area
New Caledonia	Health related data	minutes
New Caledonia	Demographic data	Count
New Caledonia	Demographic data	Count
New Zealand	Indicators of human development	Index
New Zealand	Administrative boundaries	km

Country name	Data source type	Unit
New Zealand	Demographic data	Count
New Zealand	Environmental covariates	nW/cm2/sr
New Zealand	Demographic data	Count
New Zealand	Demographic data	Count/Area
New Zealand	Health related data	minutes
New Zealand	Demographic data	Count
New Zealand	Demographic data	Count
Niue	Indicators of human development	Index
Niue	Administrative boundaries	km
Niue	Environmental covariates	nW/cm2/sr
Niue	Health related data	minutes
Marshall Islands	Indicators of human development	Index
Northern Mariana Islands	Indicators of human development	Index
Northern Mariana Islands	Administrative boundaries	km
Northern Mariana Islands	Environmental covariates	nW/cm2/sr
Northern Mariana Islands	Health related data	minutes
Palau	Indicators of human development	Index
Palau	Administrative boundaries	km
Palau	Demographic data	Count
Palau	Environmental covariates	nW/cm2/sr
Palau	Demographic data	Count
Palau	Demographic data	Count/Area
Palau	Health related data	minutes
Palau	Demographic data	Count
Palau	Demographic data	Count
Papua New Guinea	Socio-demographic data	km

Country name	Data source type	Unit
Papua New Guinea	Socio-demographic data	nW/cm2/sr
Papua New Guinea	Socio-demographic data	%
Papua New Guinea	Socio-demographic data	%
Papua New Guinea	Socio-demographic data	%
Papua New Guinea	Socio-demographic data	%
Papua New Guinea	Socio-demographic data	%
Philippines	Socio-demographic data	
Philippines	Socio-demographic data	km
Philippines	Socio-demographic data	nW/cm2/sr
Philippines		nW/cm2/sr
Philippines	Socio-demographic data	
Philippines	Socio-demographic data	
Philippines	Socio-demographic data	
Philippines	Socio-demographic data	
Philippines	Socio-demographic data	
Philippines	Socio-demographic data	
Philippines	Health related data	minutes
Philippines	Socio-economic data	
Philippines	Socio-economic data	
Philippines	Socio-economic data	
Philippines	Socio-economic data	
Fiji	Indicators of human development	
Philippines	Socio-economic data	

Country name	Data source type	Unit
Philippines	Socio-economic data	
Philippines	Socio-economic data	
Philippines	Socio-economic data	
Republic of Korea	Administrative boundaries	km
Republic of Korea	Demographic data	Count
Republic of Korea	Demographic data	Age
Republic of Korea	Environmental covariates	nW/cm2/sr
Republic of Korea	Indicators of human development	Percent
Republic of Korea	Demographic data	Count
Republic of Korea	Demographic data	Count
Republic of Korea	Health related data	minutes
Republic of Korea	Demographic data	Count
Republic of Korea	Demographic data	count
Republic of Korea	Demographic data	Count
Republic of Korea	Administrative boundaries	
Samoa	Indicators of human development	Index
Samoa	Demographic data	
Samoa	Administrative boundaries	km
Samoa	Demographic data	Count
Samoa	Environmental covariates	nW/cm2/sr
Samoa	Demographic data	Count
Samoa	Demographic data	Count
Samoa	Health related data	minutes
Samoa	Demographic data	Count
Samoa	Demographic data	Count
Republic of Korea	Indicators of human development	Index
Singapore	Indicators of human development	Index

Country name	Data source type	Unit
Singapore	Administrative boundaries	km
Singapore	Demographic data	Count
Singapore	Environmental covariates	nW/cm2/sr
Singapore	Demographic data	Count
Singapore	Demographic data	Count/Area
Singapore	Health related data	minutes
Singapore	Demographic data	Count
Singapore	Demographic data	count
Solomon Islands	Indicators of human development	Index
Solomon Islands	Socio-economic data	km
Solomon Islands	Socio-economic data	nW/cm2/sr
Solomon Islands	Socio-economic data	
Solomon Islands	Socio-economic data	
Solomon Islands	Socio-economic data	
Solomon Islands	Socio-economic data	
Solomon Islands	Socio-economic data	
Solomon Islands	Socio-economic data	
Solomon Islands	Socio-economic data	
French Polynesia	Indicators of human development	
Solomon Islands	Socio-economic data	
Solomon Islands	Administrative boundaries	
Tokelau	Indicators of human development	Index
Tokelau	Administrative boundaries	km
Tokelau	Environmental covariates	nW/cm2/sr
Tokelau	Health related data	minutes
Tonga	Socio-economic data	
Tonga	Socio-economic data	km

Country name	Data source type	Unit
Tonga	Socio-economic data	
Tonga	Socio-economic data	
Tonga	Socio-economic data	
Tonga	Socio-economic data	
Tonga	Socio-economic data	nW/cm2/sr
Tonga	Socio-economic data	
Tonga	Socio-economic data	
Tonga	Socio-economic data	
Tonga	Socio-economic data	
Tonga	Socio-economic data	
Tonga	Socio-economic data	
Guam	Indicators of human development	
Tonga		
Tonga	Socio-economic data	
Tonga	Administrative boundaries	
Tuvalu	Indicators of human development	Index
Tuvalu	Demographic data	
Tuvalu	Administrative boundaries	km
Tuvalu	Demographic data	Count
Tuvalu	Indicators of human development	asset-based wealth index that runs from 0 (no assets) to 100 (all assets)
Tuvalu	Indicators of human development	%
Tuvalu	Environmental covariates	nW/cm2/sr
Tuvalu	Indicators of human development	Percent
Tuvalu	Demographic data	
Tuvalu	Demographic data	
Tuvalu	Health related data	minutes

Country name	Data source type	Unit
Tuvalu	Demographic data	Count
Tuvalu	Demographic data	Count
Tuvalu	Administrative boundaries	
Vanuatu	Socio-economic data	
Vanuatu	Socio-economic data	km
Vanuatu	Socio-economic data	
Vanuatu	Socio-economic data	
Vanuatu	Demographic data	
Vanuatu	Socio-economic data	
Vanuatu	Socio-economic data	
Vanuatu	Socio-economic data	nW/cm2/sr
Vanuatu	Socio-economic data	
Vanuatu	Socio-economic data	
Vanuatu	Socio-economic data	
Vanuatu	Socio-economic data	
Vanuatu	Socio-economic data	
Vanuatu	Socio-economic data	
Japan	Indicators of human development	
Vanuatu		
Vanuatu	Socio-economic data	
Vanuatu	Administrative boundaries	
Viet Nam	Socio-economic data	
Viet Nam	Socio-economic data	km
Viet Nam	Socio-economic data	
Viet Nam		
Viet Nam	Socio-economic data	

Country name	Data source type	Unit
Viet Nam	Demographic data	
Viet Nam	Demographic data	
Viet Nam	Socio-economic data	
Viet Nam	Socio-economic data	nW/cm2/sr
Viet Nam	Socio-economic data	
Viet Nam	Socio-economic data	%
Viet Nam	Socio-economic data	
Viet Nam	Socio-economic data	
Viet Nam	Socio-economic data	
Viet Nam	Socio-economic data	
Viet Nam	Socio-economic data	
Viet Nam	Socio-economic data	
Kiribati	Indicators of human development	
Viet Nam		
Viet Nam	Socio-economic data	%
Viet Nam	Socio-economic data	
Viet Nam	Administrative boundaries	
Wallis and Futuna	Indicators of human development	Index
Wallis and Futuna	Administrative boundaries	km
Wallis and Futuna	Demographic data	
Wallis and Futuna	Indicators of human development	
Wallis and Futuna	Indicators of human development	
Wallis and Futuna	Environmental covariates	nW/cm2/sr
Wallis and Futuna	Health related data	minutes
Wallis and Futuna	Administrative boundaries	

Country name	Data source type	Unit
Lao People's Democratic Republic (the)	Indicators of human development	
Malaysia	Indicators of human development	
Marshall Islands	Indicators of human development	
Micronesia (Federated States of)	Indicators of human development	
Mongolia	Indicators of human development	
Nauru	Indicators of human development	
New Caledonia	Indicators of human development	
New Zealand	Indicators of human development	
Niue	Indicators of human development	
Northern Mariana Islands	Indicators of human development	
Palau	Indicators of human development	
Papua New Guinea	Indicators of human development	
Philippines	Indicators of human development	%
Republic of Korea	Indicators of human development	
Samoa	Indicators of human development	
Singapore	Indicators of human development	
Solomon Islands	Indicators of human development	
Tokelau	Indicators of human development	
Tonga	Indicators of human development	%
Tuvalu	Indicators of human development	
Vanuatu	Indicators of human development	

Country name	Data source type	Unit
Viet Nam	Indicators of human development	
Wallis and Futuna	Indicators of human development	
